# Chelerythrine triggers the prolongation of QT interval and induces cardiotoxicity by promoting the degradation of hERG channels

**DOI:** 10.1016/j.jbc.2024.108023

**Published:** 2024-11-27

**Authors:** Fang Wang, Baoqiang Wang, Xiwei Gu, Xiaoxu Li, Xinyu Liu, Baoxin Li

**Affiliations:** Department of Pharmacology, College of Pharmacy, Harbin Medical University, Harbin, Heilongjiang Province, China

**Keywords:** drug safety, cardiotoxicity, alkaloids, LQTS, lysosomal degradation

## Abstract

Cardiotoxicity is a serious adverse reaction during drug treatment. The cardiac human ether-a-go-go-related gene (hERG) channels play a crucial role in driving cardiac action potential repolarization and are a key target for drug-induced cardiac toxicity. Chelerythrine (CHE) has anticancer effects on various human cancer cells. But little is known about its drug safety currently. The purpose of this study is to explore the key mechanism of cardiac toxicity induced by CHE under pathological conditions. CHE and hypoxia prolonged QT interval and action potential duration compared with control group in guinea pigs, as measured by BL-420S biological acquisition and processing system in conjunction with optical mapping technology. hERG current was measured by patch-clamp technique, and the interaction between ubiquitin molecules and hERG channels was assessed using immunoprecipitation method at the molecular level. The colocalization of proteins and the function of lysosomes were determined *via* confocal laser scanning microscopy. Further research indicates that CHE enhances the ubiquitination process of hERG proteins by catalyzing the formation of K63 ubiquitin chains, the ubiquitination modification disrupts hERG channel homeostasis, and promotes the degradation of the channel. Mechanistically, CHE accelerates the degradation of hERG channels through lysosomes *via* HDAC6, which may easily induce cardiotoxicity caused by prolonged QT interval under hypoxic conditions.

Drug safety evaluation is an important step in drug development. With the successive discovery of some drugs that can induce acquired QT interval prolongation (long QT syndrome [LQTS), severe cases can lead to torsade de pointes or even sudden cardiac death. Therefore, the cardiotoxicity of drug-induced QT interval prolongation has become an important problem threatening drug safety (1, 2). The human ether-a-go-go-related gene (hERG) channel deficiency is the most important mechanism of drug-induced QT interval prolongation, as their unique structural characteristics determine their sensitivity to compounds with diverse chemical structures (3).

Many drugs used in clinical practice are derived directly or indirectly from natural products, which serve as crucial sources in novel drug development (4, 5), highlighting their significant role in drug research (6). Chelerythrine (CHE) is a natural benzophenanthridine alkaloid known for its antitumor, antidiabetes, and anti-inflammatory activities (7, 8). Recent studies showed that CHE can inhibit prostatic carcinoma, breast cancer, non–small-cell carcinoma, and promote apoptosis (7, 9). Although cardiotoxicity from antitumor drugs is on the rise (10, 11), research into their safety remains relatively limited. In this study, we focused on the cardiac safety of CHE and identified its main mechanism of action on the hERG channels. Considering that patients with ischemic heart disease and chronic obstructive pulmonary disease were found to have a higher likelihood of arrhythmia in clinical practice, we investigated the mechanism by which CHE inhibits hERG channels under hypoxic conditions.

Studies have shown that indole-3-propionic acid reduces oxidative stress, inflammation, and apoptosis of cardiomyocytes by inhibiting HDAC6–NOX2 signaling pathway (12). Recent studies suggest that HDAC6 is associated with atrial fibrillation (13). These studies collectively indicate that the acquisition of HDAC6 function is harmful to the heart. In our previous studies, we confirmed the binding relationship between HDAC6 and hERG channels (14), which is consistent with the results reported in the previous literature that HDAC inhibitors increase transcription of hERG channel mRNA and translation of the channel proteins (15), indicating a correlation between HDAC6 and the occurrence of arrhythmia. Moreover, it has been shown by another research that CHE targets on the hERG channels to inhibit hERG current directly (16). Valentin *et al.* (17) demonstrated that up to 40% of the hERG blockers evaluated also inhibited hERG protein trafficking. As shown in Table 1, blocking of hERG directly and inhibiting channel proteins' trafficking are equally important for drug-induced LQT. In our studies, we mainly focus on exploring the effect of CHE on the abundance of channel proteins, emphasizing sustaining action and how it affects ion channels under pathological conditions (such as hypoxia). The reduction of hERG protein leads to a decrease in the number of channels, which ultimately results in inhibiting hERG currents. The instantaneous effect of drugs on ion channels leads to a decrease in current, and hERG channel can trap blocking molecules after closure of the activation gate. Drug may be flushed out when the channel opens, leading to a potentially short-lived inhibitory effect. However, alterations in the number of ion channels can result in a relatively more persistent inhibition of the hERG current. Therefore, it is necessary to study the sustained effects of drugs on channels. Further experiments revealed that CHE affects the degradation of hERG channels through HDAC6, whereas CHE promotes the ubiquitination process of hERG proteins by catalyzing the formation of K63 ubiquitin (Ub) chains, demonstrating the ubiquitination modification disrupts the homeostasis of hERG channels. In conclusion, CHE directly blocks hERG channels and inhibits channel protein trafficking, suggesting a higher risk of CHE-induced LQTS.

**Table 1:** Drugs that inhibit hERG channel protein trafficking and induce LQTS

Drug	hERG channel blocking	hERG channel inhibition	QT-action potential duration (APD) increasing	Reference
Arsenic trioxide	+	++	Yes	Benoit *et al.* (2004) (52), Eckhard *et al.* (2004) (53)
Celastrol	+	++	Yes	Haiyan *et al.* (2006) (54)
Digitoxin/ouabain/digoxin	－	+	Yes	Lu *et al.* (2007) (55)
Dofetilide	+	Not reported	Yes	Carmeliet *et al.* (1992) (56)
Fluoxetine	+	+	Yes	Rajamani *et al.* (2006) (57), Barbara *et al.* (2005) (58)
Ketoconazole	+	+	Yes	Dumaine *et al.* (1998) (59), Takemasa *et al.* (2007) (60)
Pentamidine	－	+	Yes	Jason *et al.* (2005) (61)
Probucol	－	+	Yes	Jun *et al.* (2007) (62)
Thioridazine	++	+	Yes	Benoit *et al.* (2004) (52), Barbara *et al.* (2005) (58)

## Results

### The effects of CHE and hypoxia on QT interval and action potential duration in guinea pigs were measured at animal level

**Figure 1:**
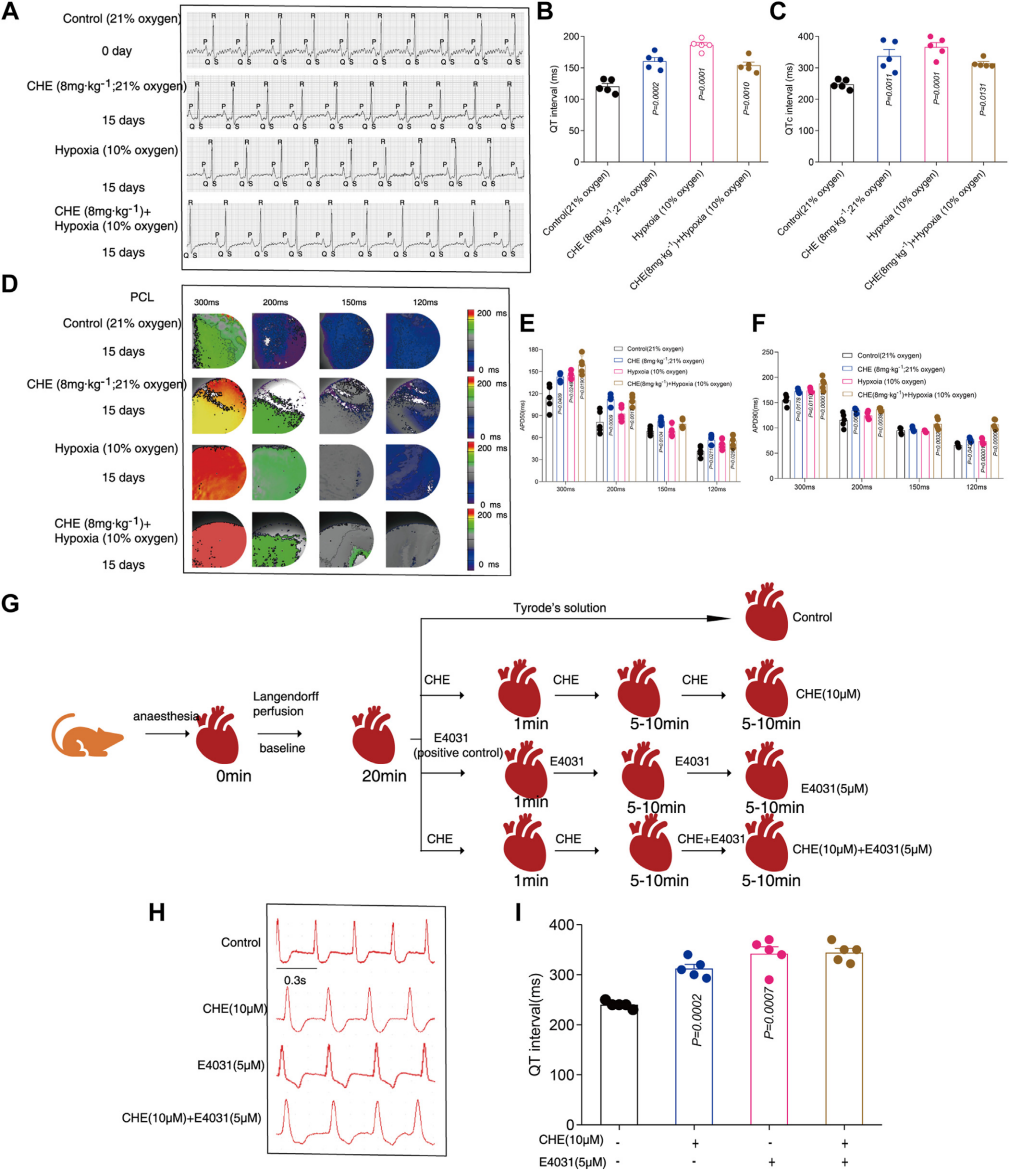
**The effects of CHE and hypoxia on QT interval and action potential duration (APD) in guinea pigs were measured at animal level.***A*, electrocardiogram of guinea pigs. *B* and *C*, effects of CHE and hypoxia on QT/QTc interval in guinea pigs (n = 5). *D*, representative map of optical mapping of guinea pigs. *E* and *F*, effects of CHE and hypoxia on APD50 and APD90 in guinea pigs (n = 5). Shared control and CHE (8 mg kg^−1^) group with Fig. S2. *G*, schematic diagram of experimental design. *H* and *I*, representative and statistical results of QT interval (n = 5). The data presented here were representative of five independent experiments. Error bars were represented as mean ± SD, ordinary one-way ANOVA was used for significance test, ∗*p* < 0.05, ∗∗*p* < 0.01, and ∗∗∗*p* < 0.001. CHE, chelerythrine.

First, BL-420S biological acquisition and processing system in conjunction with optical mapping technology were used to detect the effects of CHE and hypoxia on QT interval and action potential duration (APD) in guinea pigs. CHE (8 mg kg^−1^ or 16 mg kg^−1^) and hypoxia prolonged QT interval compared to the control group in guinea pigs (Figs. 1, *A*–*C*, S2, *A*–*C*). There were no significant statistical differences between CHE (8 mg kg^−1^) or hypoxia alone compared to CHE (8 mg kg^−1^) + hypoxia (Fig. 1, *A*–*C*). The QT interval of CHE (16 mg kg^−1^) and CHE (8 mg kg^−1^) + hypoxia group was not continuously prolonged. However, CHE (8 mg kg^−1^) + hypoxia increases the mortality rate of guinea pigs (Fig. S1). Subsequently, the guinea pig hearts were paced at cycle lengths of 120, 150, 200, and 300 ms, respectively, whereas optical AP curves were recorded. Both CHE and hypoxia prolonged the APD of guinea pigs under different cycle length pacing (Fig. 1, *D*–*F*). However, there was no significant difference between CHE (16 mg kg^−1^) and CHE (8 mg kg^−1^) regarding their effect on the APD in guinea pigs (Fig. S2, *D*–*F*). These results are consistent with the results of QT interval in guinea pigs.

Considering that the cardiac ventricular action potential depends on voltage-gated ion channels, provided evidence that compared with the CHE group alone, there was no significant change in the coincubation group of CHE + E4031 (E4031, an antiarrhythmic small molecule, selectively inhibits hERG in guinea pig and rabbit ventricles (18)) in the QT interval (Fig. 1, *G*–*I*), indicating that CHE exerts a strong inhibitory effect on hERG channels, which is an important factor contributing to the prolongation of the QT interval caused by CHE treatment.

### The effects of CHE and hypoxia on hERG channel proteins and currents in HEK293-hERG cells

**Figure 2:**
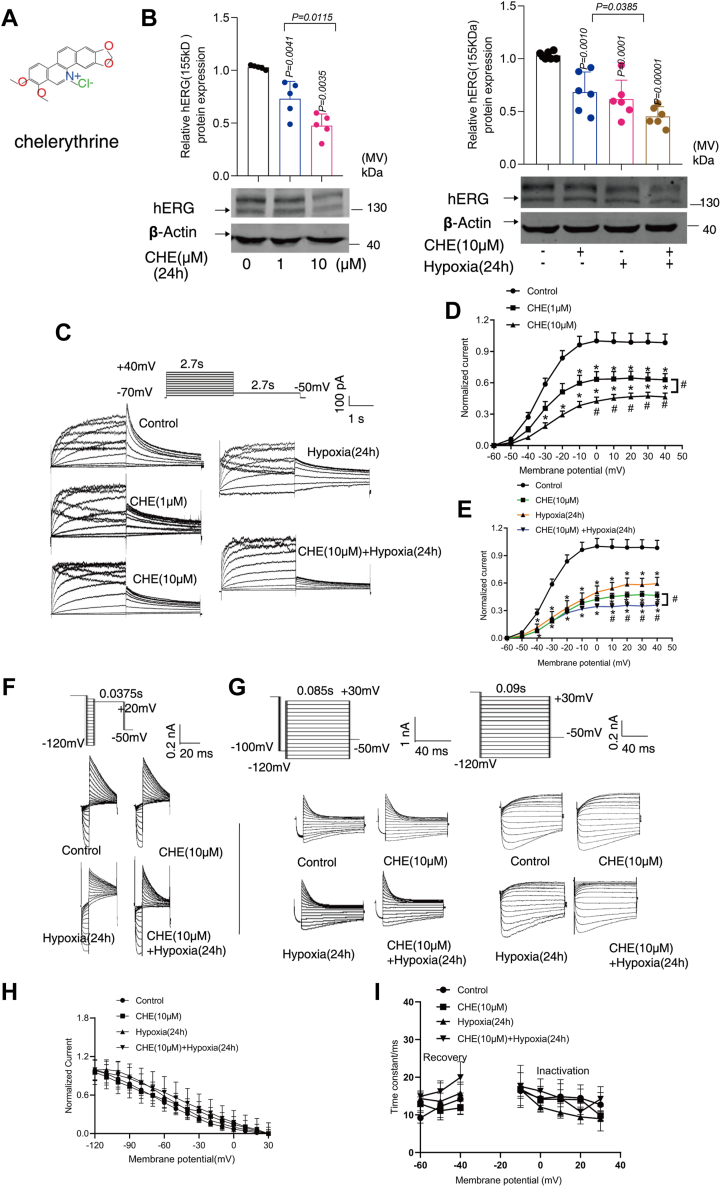
**The effects of CHE and hypoxia on hERG channel proteins and currents in HEK293–hERG cells.***A*, the structure of the drug. *B*, the effect of CHE on the expression of hERG channel protein. *C*, the voltage-clamp protocol and representative hERG current traces. *D* and *E*, I–V curve of the tail current density was the tail current normalized to the capacitance of each cell and maximal current amplitude of control group (n = 11). *E*, ∗*p* < 0.05 compared with their corresponding control (0 μM group), #*p* < 0.05 compared with their corresponding CHE incubation group (10 μM group). *F*, voltage-clamp protocol and representative current tracing for steady-state inactivation. *G*, voltage-clamp protocol and representative current tracing for onset of inactivation (*left*) and recovery from inactivation (*right*). *H*, normalized steady-state inactivation curves (n = 4). *I*, the time constant for inactivation and recovery (n = 4). The hERG activation and inactivation–voltage relationships were fitted with a Boltzmann function, y = 1/{1 + exp [(V_1/2_ − V)/k]}, where y is the normalized peak conductance, V_1/2_ is the half-activation or half-inactivation potential, V is the variable (test) voltage, and k is the slope factor. The data presented here were representative of a minimum of three independent experiments. Error bars were represented as mean ± SD; two-tailed Student′s *t* test and ordinary one-way ANOVA were used for significance test, ∗*p* < 0.05, ∗∗*p* < 0.01, ∗∗∗*p* < 0.001. CHE, chelerythrine; hERG, human ether-a-go-go-related gene.

The structure of CHE is shown in Fig. 2*A*. To assess the impact of CHE and hypoxia on hERG channels, we performed Western blot assays and found that CHE significantly inhibited hERG protein expression in a concentration-dependent manner. Moreover, hypoxia conditions augmented the inhibition of hERG channels induced by treatment of CHE for 24 h (Fig. 2*B*). The result from whole-cell patch-clamp techniques also showed that CHE exerts a concentration-dependent inhibitory effect on hERG current. In addition, the inhibition effect of CHE on hERG channels was enhanced under hypoxia conditions (Fig. 2, *C*–*E*).

Afterward, the effects of CHE, hypoxia, and CHE combined with hypoxia incubation for 24 h were examined on the steady-state inactivation and instantaneous inactivation of hERG channels. There was no significant change in the half-activation potential (V_1/2, act_) and half-inactivation potential (V_1/2, inact_) of CHE and hypoxia (control group: V_1/2,act_ was −22.42 ± 1.52 mV; V_1/2,inact_ was −55.58 ± 6.04 mV, CHE [10 μM] group: V_1/2,act_ was −24.24 ± 2.94 mV; V_1/2,inact_ was −55.74 ± 2.61 mV, hypoxia [24 h] group: V_1/2,act_ was −22.45 ± 4.56 mV; V_1/2,inact_ was −58.65 ± 2.32 mV, CHE [10 μM] + hypoxia [24 h] group: V_1/2,act_ was −24.00 ± 3.04 mV; V_1/2,inact_ was −57.12 ± 7.53 mV), indicating that CHE and hypoxia did not significantly change voltage-dependent activation and inactivation. Both CHE and hypoxia had little or no effect on steady-state inactivation (Fig. 2, *F* and *H*), onset of inactivation, and recovery from inactivation (Fig. 2, *G* and *I*). In other words, CHE and hypoxia have limited influence on the gating characteristics of hERG channels, while the inhibition of current may be attributed to a reduction in the number of channels. These main current properties are similar to the previously published and relevant literature (19, 20, 21).

### Identify the degradation pathway of hERG channel inhibition by CHE in HEK293-hERG cells and neonatal rat cardiomyocytes

**Figure 3:**
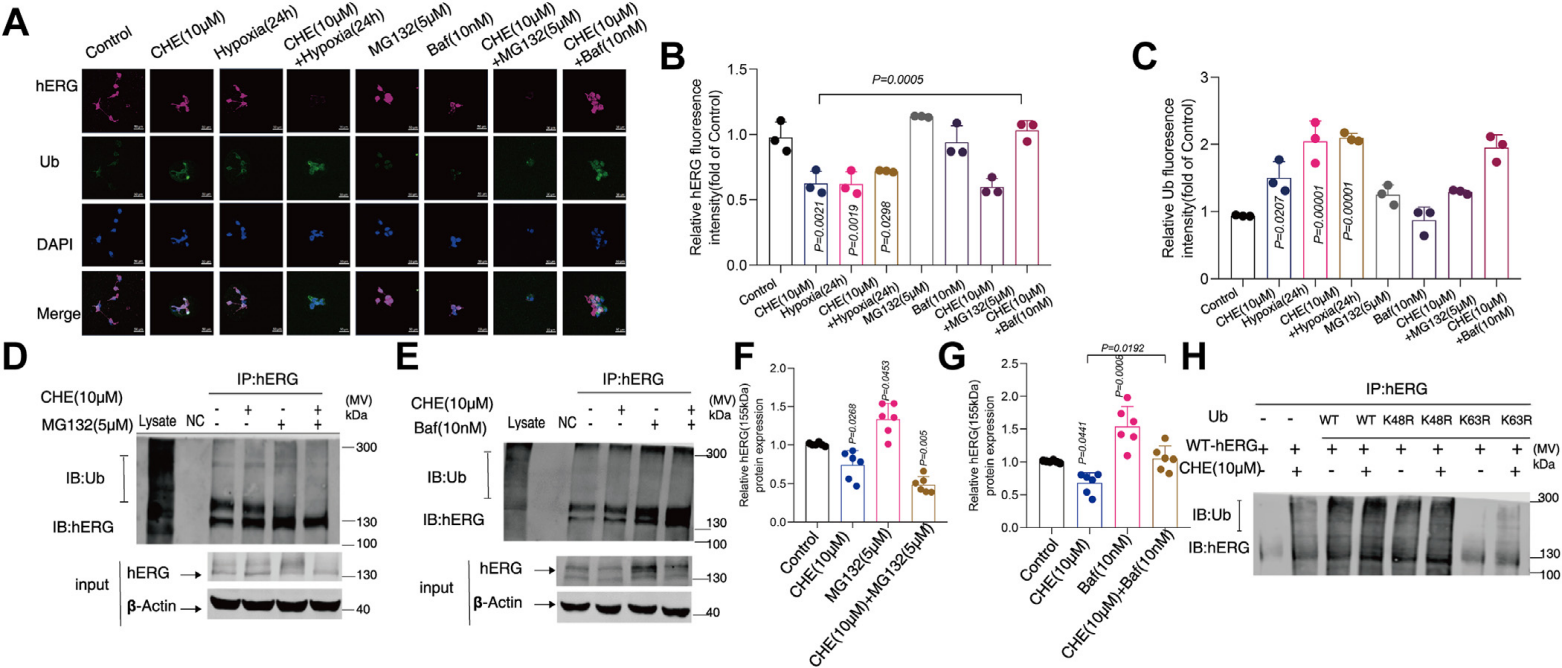
**Identify the degradation pathway of hERG channel inhibition by CHE in HEK293–hERG cells and neonatal rat cardiomyocytes (NRCMs).***A*–*C*, microscopic representation of hERG channels and ubiquitin molecules. Confocal images of cells stained with hERG antibodies (*magenta*); confocal laser scanning microscopy images of cells stained by Ubiquitin antibodies (*green*); 4′,6-diamidino-2-phenylindole (nucleus, *blue*); scale bar represents 50 μm, n = 3. Proteasome inhibitor MG132 does not affect the (*D*) hERG ubiquitination (n = 4) and (*F*) protein content (n = 6) changes and lysosomal inhibitor Baf increased (*E*) hERG channel ubiquitination modification (n = 4) and restored (*G*) hERG protein expression levels (n = 6) caused by CHE by Western blot. *H*, CHE regulates the expression of hERG protein through K63-linked polyubiquitination (n = 4). The data presented here were representative of a minimum of three independent experiments. Error bars were represented as mean ± SD, ordinary one-way ANOVA was used for significance test, ∗*p* < 0.05, ∗∗*p* < 0.01, ∗∗∗*p* < 0.001. CHE, chelerythrine; hERG, human ether-a-go-go-related gene.

Proteasome inhibitor MG132 and lysosome inhibitor bafilomycin A1 (Baf) were added to detect the expression of hERG channels. Coimmunoprecipitation and confocal laser scanning microscopy results showed that CHE and hypoxia can promote the binding of Ub molecules to hERG channels (Fig. 3, *A*–*E*). The ubiquitination level of the hERG channels did not show significant changes in the coincubation group with proteasome inhibitor MG132 compared with CHE single incubation group, and MG132 could not restore the decreased hERG protein expression caused by CHE (Fig. 3, *D* and *F*). However, lysosome inhibitor Baf could restore the decrease of hERG protein expression and increase the ubiquitination level of hERG channels by CHE (Figs. 3, *E* and *G*, S4, *A*, *B* and *E*).

Ubiquitin (Ub) can be polyubiquitinated to substrates at lysine sites, and previous research has highlighted the significant role of Ub chain formation in substrate degradation. It is widely accepted that the formation of K48 Ub chains is mainly related to proteasome degradation, whereas the formation of K63 Ub chains is related to lysosomal degradation (22, 23). K48 and K63 of Ub were mutated to investigate the impact of CHE on the types of Ub chains on hERG channels, thereby evaluating the effect of CHE on the multiubiquitination of hERG channels. The results indicated that only K63R Ub interrupted the polyubiquitination of hERG channels under the presence of CHE, without affecting the formation of K48R Ub chains (Fig. 3*H*).

In summary, the aforementioned evidence strongly indicated that CHE regulates hERG protein expression by facilitating K63-linked polyubiquitination, leading to the degradation of hERG channels mediated by lysosomes.

### The regulation of hERG channel protein expression by histone deacetylase HDAC6 was determined

**Figure 4:**
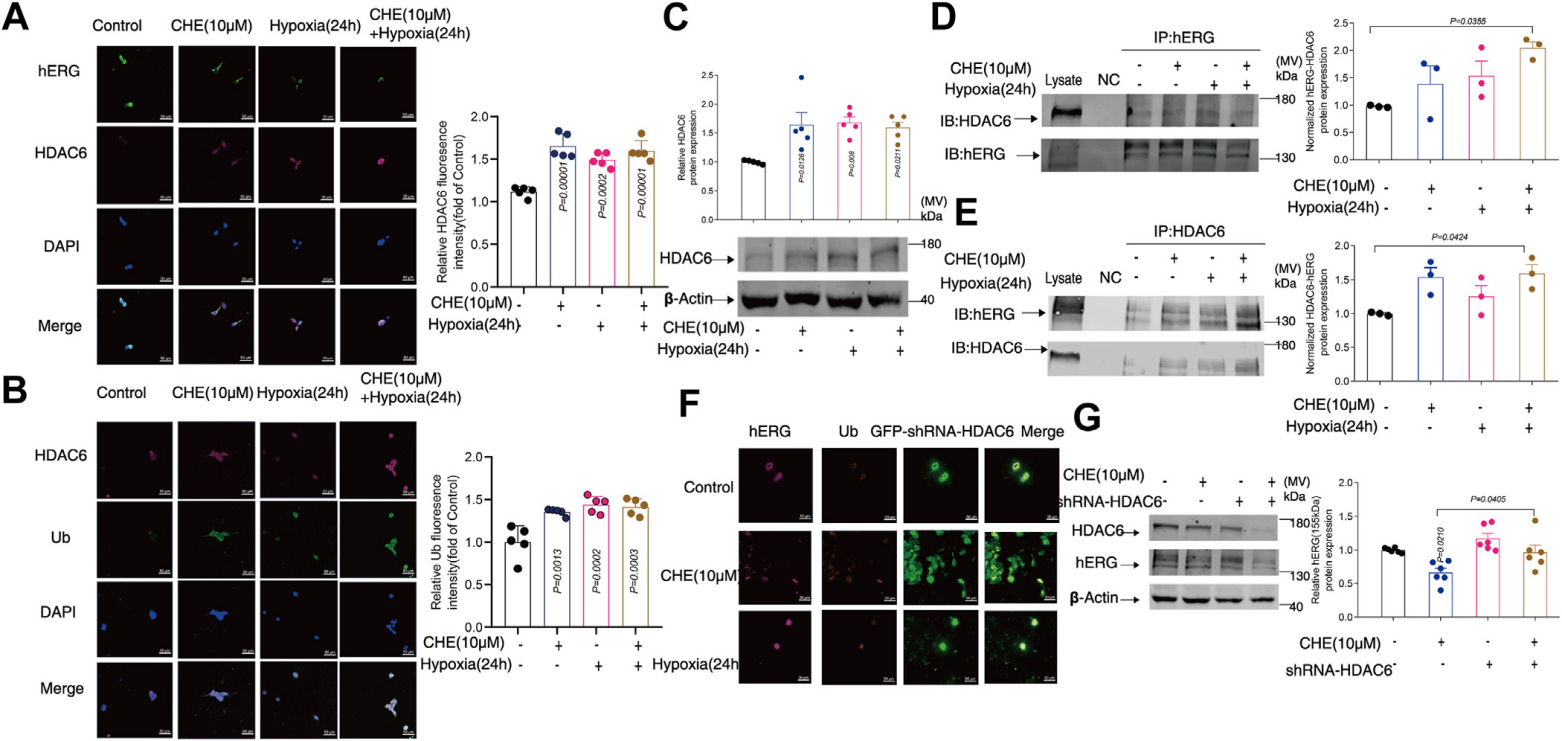
**The regulation of hERG channel protein expression by histone deacetylase HDAC6 was determined.***A* and *B*, colocalization of hERG channels with histone deacetylase HDAC6 and ubiquitin (Ub) molecules. Confocal images of cells stained with HDAC6 and hERG antibodies (*top*) and confocal images of cells stained with HDAC6 and Ub antibodies (*bottom*), scale bar represents 50 μm, n = 5. *C*, the effects of CHE and hypoxia on HDAC6 protein expression (n = 5). Western blot representation and statistical results of (*D*) hERG protein primary antibody precipitation HDAC6 or (*E*) HDAC6 protein primary antibody precipitation hERG (n = 3). Microscopic representation of (*F*) hERG channels and Ub molecules and (*G*) expression of hERG channel protein knockdown of HDAC6 (n = 6). Confocal images of cells stained with hERG antibodies (*magenta*); confocal laser scanning microscope image of cells stained with Ub antibodies (*orange*); GFP-shRNA-HDAC6 (*green*), scale bar represents 50 μm, n = 3. The data presented here were representative of a minimum of three independent experiments. Error bars were represented as mean ± SD, ordinary one-way ANOVA was used for significance test, ∗*p* < 0.05, ∗∗*p* < 0.01, ∗∗∗*p* < 0.001. hERG, human ether-a-go-go-related gene.

Studies have shown that the zinc finger domain located in the C-terminal region of HDAC6 is very important for Ub–protein interactions. Since the Ub–proteasome pathway is unable to degrade ubiquitinated protein aggregates effectively, one of the main cellular mechanisms for clearing ubiquitinated protein aggregates is through the action of HDAC6, which degrades protein aggregates following the formation of microtubule organization center aggregates (24). Confocal laser scanning microscopy results show that HDAC6 and hERG channels are located at the same location (Fig. 4*A*) and subsequent immunocoprecipitation experiments revealed that there is indeed an interaction between HDAC6 and hERG channels (Figs. 4, *D* and *E*, S4, *C* and *D*). While, CHE and hypoxia increased HDAC6 protein expression (Fig. 4*C*) and the expression of HDAC6–hERG protein complex under hypoxia conditions (Fig. 4, *D* and *E*). The fluorescence of Ub molecules was enhanced synchronously with the increase of HDAC6 fluorescence under the conditions of CHE and hypoxia (Fig. 4*B*). Furthermore, under the presence of CHE and hypoxia conditions, the labeling intensity of Ub molecules was largely diminished (Fig. 4*F*), while the protein expression of hERG channels was largely restored (Figs. 4*G*, S3, *A* and *B*) following the knockout of HDAC6. Collectively, these results indicate that CHE and hypoxia can regulate the expression of hERG channels by regulating the ubiquitination process of hERG channels through HDAC6.

### The effects of CHE and hypoxia on the number and function of lysosomes

**Figure 5:**
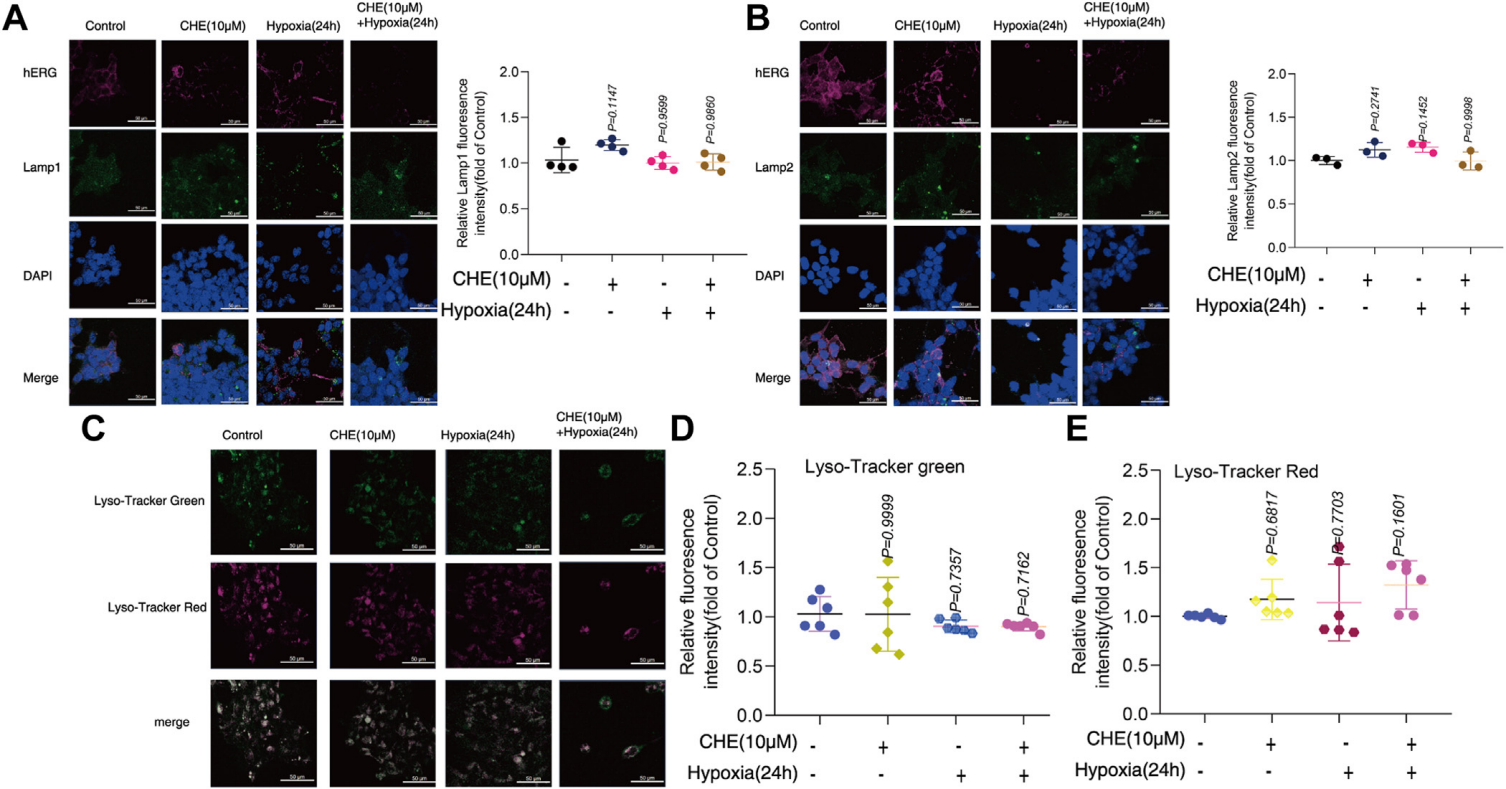
**The effects of CHE and hypoxia on the number and function of lysosomes.***A* and *B*, confocal micrograph of hERG channels and lysosome markers. LAMP1 (lysosome marker, *left*), n = 4, LAMP2 (lysosome marker, *right*), n = 3, scale bar represents 50 μm. *C*–*E*, effect of CHE and hypoxia on lysosome acidity. (*Green*: Lyso-Tracker *Green*, *Red*: Lyso-Tracker *Red*, scale bar represents 50 μm, n = 6). The data presented here were representative of a minimum of three independent experiments. Error bars were represented as mean ± SD, ordinary one-way ANOVA was used for significance test, ∗*p* < 0.05, ∗∗*p* < 0.01, ∗∗∗*p* < 0.001. CHE, chelerythrine; hERG, human ether-a-go-go-related gene; LAMP, lysosome-associated membrane protein.

Lysosome-associated membrane protein 1 (LAMP1) is a highly glycosylated glycoprotein mainly present on the lysosomal membrane, which is involved in various physiological and pathological processes in the cells (25, 26). Lysosomal-associated membrane protein-2 (LAMP2) is a fairly abundant lysosomal glycoprotein that acts as a receptor for proteins introduced into lysosomes directly (27). The colocalization of hERG with lysosome-related proteins LAMP1 and LAMP2 was detected by confocal laser scanning microscopy (Fig. 5, *A* and *B*). Lyso-Tracker Red and Lyso-Tracker Green can be selectively retained in more acidic lysosomes to achieve specific fluorescent labeling of lysosomes. After that, Lyso-Tracker Red and Lyso-Tracker Green-labeled lysosomes were used to detect the acidity of lysosomes. Cells treated with CHE and hypoxia for 24 h did not exhibit any changes in lysosomal acidity (Fig. 5, *C*–*E*), suggesting that CHE and hypoxia did not affect lysosomal homeostasis.

### PKC inhibitor bisindolylmaleimide I was incubated for 24 h to determine the expression of related proteins

CHE is known as a PKC inhibitor. The results showed that bisindolylmaleimide I (BIM-1) (a potent selective inhibitor of PKC) (3 μM) reduced the expression of HDAC6 (Fig. S5*B*), which was consistent with the results reported in the literature that PKCα activates HDAC6 expression (28). BIM-1 (1 μM and 3 μM) inhibits hERG protein (Fig. S5*A*). However, BIM-1 did not affect the ubiquitination level of hERG channels (Fig. S5*C*). These results indicate that the reduction of hERG channel proteins induced by PKC inhibitor BIM-1 is not achieved through ubiquitination degradation pathway, and the PKC pathway is not related to the lysosomal degradation of hERG channels mainly presented in this article.

## Discussion

The QT interval is the time between ventricular depolarization and repolarization. Drug-induced QT prolongation is usually caused by blocking potassium channels encoded by the hERG gene, resulting in cardiac toxicity (3, 29). The hERG potassium channels is a tetramer composed of four subunits and is related to cardiac repolarization mainly. Structurally diverse compounds have been shown to inhibit hERG ion channels and exhibit cardiotoxicity associated with hERG channels. Many drugs from different therapeutic fields have been withdrawn from the market or restricted strictly because of cardiotoxicity associated with hERG channels, such as astemizole, cisapride, terfenadine, dofetilide, trovafloxacin, vardenafil, and ziprasidone (30, 31). Since the early 2000s, early assessment of hERG-associated cardiotoxicity has been a crucial step in drug development, given the severity of cardiac events related to hERG inhibition. Acquired LQTS can be caused by drugs or diseases, such as myocardial ischemia and hypoxia, heart failure, and so on. Myocardial ischemia, accompanied by hypoxia and resulting in QT interval prolongation, has become a common clinical symptom and is an important factor in sudden cardiac death. Therefore, fully understanding the effects of drugs on hERG channels and discovering the characteristics of drug-inhibiting hERG channels is of great significance for reducing the risk of drug-induced cardiac arrhythmias associated with hERG deficiency or channel blocking, optimizing drug design to minimize adverse effects, and guiding clinical rational drug use (32, 33).

At present, with the continuous increase in the application of traditional Chinese medicine, many drugs have shown satisfactory effects in treating diseases, such as cardiovascular diseases, cerebrovascular diseases, neurodegenerative diseases, and cancer (34). However, toxic reaction of drugs often manifest after long-term use since many traditional Chinese medicines containing alkaloids are prone to causing adverse reactions. For example, aconitine acts on the heart to produce abnormal excitability directly (35). Ephedrine acting on the heart can cause cardiac depression, resulting in toxic effects (36). Matrine mainly acts on the central nervous system, which can excite and then paralyze the central nervous system (37, 38). In previous studies, we found that alkaloids tend to inhibit hERG channels, leading to cardiotoxicity with prolonged QT interval in guinea pigs. Example of such alkaloids include berberine (39), rutaecarpine (40), and so on. In the current research, the main focus was to investigate the effect of the alkaloid CHE on the cardiac hERG channels. CHE promotes hERG channel degradation, resulting in a decrease in membrane stable protein expression and a prolongation of QT interval in guinea pigs. CHE at a concentration of 8 mg kg^−1^ was chosen based on the clinical dosage recorded in the Chinese Pharmacopoeia (Edition 2020) (conversion from human to animal dosage) (41). We provided evidence that CHE (8 mg kg^−1^ or 16 mg kg^−1^) significantly prolonged QT interval compared to the control group in guinea pigs. However, compared with the CHE (16 mg kg^−1^), CHE (8 mg kg^−1^) did not show statistically significant difference, suggesting that CHE might not have a dose-dependent effect on the prolongation of QT interval in guinea pigs. CHE (8 mg kg^−1^) + hypoxia prolonged the QT interval compared with the control group, but there was no statistical significance compared with the CHE and hypoxia group alone. Statistically, there was no further prolongation of the QT interval, which is probably because of the compensation occurred in animals. However, CHE (8 mg kg^−1^) combined with hypoxia remarkably increases the mortality rate of guinea pigs, which strongly suggested that the toxicity of CHE is enhanced under hypoxic conditions. Many studies have investigated the role of CHE against different types of cancer. CHE is a multitargeted agent with broad-spectrum antitumor effects. But its cardiotoxicity cannot be ignored, the off-target inhibition of the hERG potassium current has been, and still is, a significant secondary pharmacology liability in small-molecule drug discovery and development. Tends to use thresholds closer to 30-fold based on assumed clinical concentrations when the hERG characterization was completed prior to first human testing. Some drugs may also only have exhibited QTc prolongation at a supratherapeutic level (42). The therapeutic dosage of CHE (43, 44) is comparable to the dosage of cardiotoxicity caused by hERG inhibition-induced acquired LQTS. Therefore, it is extremely necessary to pay attention to changes in QT interval in order to use drugs reasonably and avoid risks.

In the current study, the evidence presented previously reveals that hERG channels were significantly inhibited by the treatment of CHE, which could be substantially enhanced under pathological conditions (hypoxia) without influence on the gating characteristics of hERG channels. Subsequently, results of immunoprecipitation and amino acid mutation show that CHE accelerates the degradation of hERG channels through the lysosomal pathway. CHE regulates the expression of hERG protein by catalyzing polyubiquitination of K63 connections, and the formation of K63 Ub chains is facilitating its transport to and degradation within the lysosome. This coordinated process highlights the intricate regulatory network governing hERG channel stability in the presence of cardiotoxic drugs or pathological conditions.

**Figure 6:**
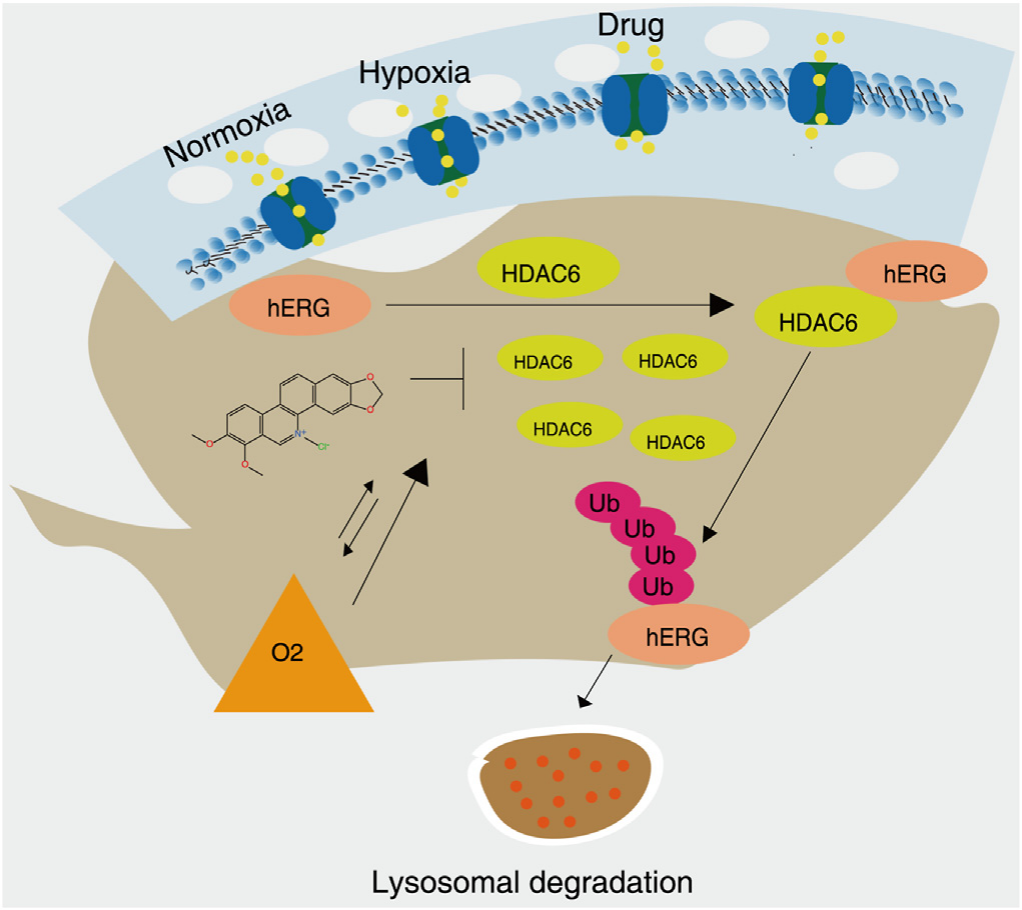
**The lysosomal degradation mechanism of drug inhibiting hERG channels.** hERG, human ether-a-go-go-related gene.

Post-translational modification of proteins plays an important role in the occurrence and development of diseases. Ubiquitination modification is not conducive to maintaining hERG channel homeostasis in this study. It has been to report that HDAC6 contains a Ub-binding domain that aggregates multiubiquitinated proteins into the kinetin complex, allowing them to be isolated into the microtubule organization center in the perinuclear region to form aggregates (24, 45). HDAC6 is mainly localized in the cytoplasm (46) and is Ub dependent, playing a crucial role in regulating protein degradation (47). CHE and hypoxia can capable of enhancing the expression of HDAC6. In constant, the knockdown of HDAC6 can significantly reduce the level of Ub of hERG channels and simultaneously lead to an increase in the expression of hERG channels. Considering the ubiquitination is an important signaling molecule for the degradation of membrane proteins into lysosomes, we supposed that CHE and hypoxia caused the degradation of ubiquitinated hERG channels in lysosomes *via* HDAC6. Results from confocal laser scanning microscopy showed that hERG channels and lysosomal signature proteins (LAMP1 and LAMP2) to locate their positions in the cytoplasm (48, 49), and then CHE and hypoxia not exhibit any changes in lysosomal acidity. The detailed molecular mechanism is shown in Fig. 6. CHE and hypoxia did not affect the number and function of lysosomes, which provides a basis for the degradation of ubiquitinated hERG channels.

In summary, the effects of CHE on facilitating channel degradation and inhibiting hERG channel protein are significantly increased under pathological conditions such as hypoxia. The synergistic regulatory mechanism underlying this enhanced inhibition lies in the fact that drugs and hypoxia greatly strengthen the activation of deacetylase HDAC6, which promotes the degradation of ubiquitinated hERG protein within lysosomes. These comprehensive results suggest that drugs may easily cause potential cardiotoxicity under hypoxic conditions, which highlights the necessity to avoid cardiotoxicity of certain drugs under pathological conditions.

## Experimental procedures

### Animals

Guinea pigs (male, weight 320–350g, 6–8 weeks old) were purchased from Liaoning Changsheng Biotechnology Co, Ltd and were reared in standard animal room conditions (temperature 21–23 °C; humidity 55–60%) for 3–5 days. All animal samples were carried out in a blinded manner. CHE (8 mg kg^−1^ or 16 mg kg^−1^) was intraperitoneally administered every day. To induce hypoxia, guinea pigs were placed in a Plexiglas chamber (830∗580∗510 mm) with an FiO_2_ of 10%, adjusted with an oxygen monitor connected to an electronic valve (Hangzhou Ai-Pu Instrument Equipment Co, Ltd), which uses nitrogen to displace oxygen from the chamber. This FiO_2_ generates a stable arterial partial pressure of O_2_ of approximately 35 mm Hg (50, 51). Control was placed into the Plexiglas chamber containing room air (FiO_2_:21%) for the experiments. All animals remained in the chamber for 15 days where they had access to Purina Laboratory Chow and water ad libitum.

### Electrocardiogram determination

Prior to grouping the guinea pigs, electrocardiograms were conducted. The QT interval measured at this time served as the starting value. (This means that each experimental group has a baseline value before CHE or hypoxia intervention as their self-control). The animals were then randomly divided into the CHE group, hypoxia group, and CHE + hypoxia group. The QT intervals for each experimental group were measured 15 days later, with the comparison made against their own state before being subjected to drug treatment or hypoxia.

### APD determination

Guinea pigs were anesthetized, and their hearts were gently excised for APD measurement. The animals were randomly divided into the control group, CHE group, hypoxia group, and CHE + hypoxia group. The APD for each experimental group was measured 15 days later.

### Cell culture

HEK293-hERG cells (Research Resource Identifier [RRID]: CVCL_C0RT) were kindly donated by the Montreal Heart Institute of Canada. HEK293-hERG cells were maintained in DMEM containing 10% fetal bovine serum (FBS; Gibco; 16140089), 1% penicillin–streptomycin, and 400 μg·L^−1^ G418 in a 5% CO_2_ atmosphere at 37 °C.

### DNA constructs of HEK293-hERG cells

hERG complementary DNA was subcloned into BamHI–EcoRI sites of the pCDNA3 vector. This vector contains a cytomegalovirus promoter and an SV40 promoter, which drive the expression of the inserted complementary DNA (hERG) and neomycin-resistant gene, respectively. The HEK-293 cells were transfected with this construct by a calcium phosphate precipitate method or a lipofectamine method. After selection in 800 g/ml geneticin (G418; Gibco) for 15 to 20 days, single colonies were picked with cloning cylinders and tested for HERG current. The stably transfected cells were cultured in DMEM supplemented with 10% FBS and 400 g/ml G418.

### Construction of hypoxic cell model

DMEM low-sugar medium and 1640 medium were prepared 1:1 when the cell density reaches 70%–80%. The cells with good growth condition were taken out of the incubator (37 °C, 5% CO_2_), and the culture medium in the bottle was discarded and replaced with the serum-free hypoxic culture medium. Finally, the cells replaced with the culture medium were placed in a three-gas incubator (37 °C, 5% CO_2_, 94% N_2_, and 1% O_2_) for 24 h to establish a hypoxic cell model.

### Langendorff perfusion of isolated heart and QT recording

Isolated heart Langendorff perfusion and QT recording were performed as previously described (18). Briefly, the isolated hearts were connected to a cardiac perfusion apparatus through the aorta and were perfused with Tyrode’s solution. The composition of Tyrode’s solution was (in millimolar) NaCl 125, KCl 4.5, NaH_2_PO_4_ 1.8, NaHCO_3_ 24, CaCl_2_ 1.8, MgCl_2_ 0.5, and dextrose 5.5 in deionized water. The experimental procedure is briefly described as follows: First, the isolated hearts were allowed to equilibrate for a minimum of 20 min to ensure their stability in a pilot study where no pharmacological intervention was applied. Subsequently, the Tyrode’s solution was replaced with E4031 solution (5 μM, in Tyrode’s solution) and CHE solution (10 μM, in Tyrode’s solution) continued to be infused into the heart for 1 min. Finally, the heart was perfused with CHE (10 μM) together with E4031 (5 μM) mixture solution to observe the changes.

### Neonatal rat cardiomyocyte isolation, culture, and treatment

Based on the guidelines and approval of the Animal Ethics Experimentation Committee of Harbin Medical University, neonatal rat cardiomyocytes were dissociated from the 1-day-old male Sprague–Dawley rat heart. After the heart tissues were quickly harvested and placed in ice-cold PBS to remove blood, they were trimmed to tissue blocks and digested at room temperature with 0.25% trypsin (T1300; Solarbio). The digested neonatal rat cardiomyocytes were seeded in a 6-well culture dish at a density of 1 × 10^5^ per cm^2^ and cultured in DMEM/F12 (11330032; Gibco) containing 15% FBS in a 5%CO_2_ humidified incubator at 37 °C for 24 h to carry out the following experiments.

### Materials and reagents

CHEs (HPLC ≥98%) were obtained from the National Institutes for Food and Drug Control; MG132 and bafilomycin A1 were obtained from the Sigma–Aldrich, RH237 were obtained from the Invitrogen, BFA were obtained from the Beyotime. These compounds were dissolved in dimethyl sulfoxide to yield a stock concentration, and the dimethyl sulfoxide content in the final concentration of the experiment was less than 1‰.

### Plasmid transfection

The target gene interference plasmids shRNA-HDAC6 (sequence targeted human HDAC6 (5′-CGGTAATGGAACTCAGCACAT-3′)) and CMV enhancer-MCS-3flag-polyA-EF1A-zsGreen-sv40-puromycin K48R-Ub, K63R-Ub, WT-Ub were purchased from by Shanghai Gene Co, Ltd. Cells were plated into 5 × 10^5^ cells/well and inoculated into 6-well plate in 2 ml medium. After cells reached 60%–70% confluence, plasmid transfection was performed with Lipofectamine 2000 (ThermoFisher, catalog no.: 11668019) in Opti-MEM. Cells were gathered at 48 h post-transfection and used for further experiments.

### Western blotting

After drug or hypoxia treatment, cells were harvested by scraping and washed two to three times with PBS, then lysed with RIPA Lysis Buffer (Beyotime, P0013D). The concentration of protein in the supernatant was determined by BCA protein assay kit. Equal amounts of proteins were separated by SDS–polyacrylamide gels and then electroblotted onto nitrocellulose membranes. After blocking with 5% skim milk, the membranes were incubated with primary and secondary antibodies, respectively. The final immunoblot was subjected to fluorescence scanning using the Odyssey system. The source of the antibodies involved in the experiment was as follows. hERG (1:200 dilution; Santa Cruz Biotechnology, sc-377388, RRID: AB_3107179), HDAC6 (1:1000 dilution, Cell Signaling Technology, catalog no.: 7612, RRID: AB_10889735), β-actin (1:1000 dilution, ABclonal, catalog no.: AC004, RRID: AB_2737399), Ub (1:200 dilution, Santa Cruz Biotechnology, sc-8017, RRID: AB_628423).

### Immunoprecipitation assay

The cells treated with drugs and hypoxia were collected, and the cells were lysed with lysis buffer on ice for 30 min. The cell lysates were incubated with the designated antibodies and placed in a vertical mixer at 4 °C overnight. A total for 200–400 μg of protein samples from cells were incubated with 2 μg antibody or IgG. After adding magnetic agarose beads, the mixture was kept in a vertical mixer at 4 °C overnight. Finally, the immunoprecipitation was washed three times with Tris-buffered saline with Tween-20, and then SDS-PAGE Loading Buffer was added to elute the complexes. Finally, immunoblotting was performed to analyze the precipitated proteins.

### Patch-clamp data detection

The glass microelectrode was drawn by an electrode drawing apparatus, and different electrode liquids were poured into the glass microelectrode according to the signals to be detected. After removing the air bubbles, the electrode was fixed on the silver wire probe of the patch-clamp micromanipulation device. After lowering the microelectrode to the liquid surface, zero the baseline to remove the water entry resistance and compensate for the electrode tip potential. Slowly move the microelectrode to contact the cell membrane surface and apply negative pressure until a GΩ-level high-resistance seal is formed. At this time, the membrane was quickly aspirated to form a whole-cell patch-clamp configuration, and the negative pressure was removed. According to the specified stimulation program, the action potential was recorded in the current clamp mode, or the channel current was recorded in the voltage clamp mode. The external solution for recording K^+^ currents contained (in millimolar) NaCl 136, KCl 5.4, MgCl_2_.6H_2_O 1, CaCl_2_ 1.8, Hepes 5, and glucose 10 (pH 7.37 with NaOH). The pipette solution for recording K^+^ currents contained (in millimolar) KCl 130, Hepes 10, MgCl_2_.6H_2_O 1, EGTA 5, Mg–ATP 5, and GTP 0.1(pH 7.20 with KOH).

### Immunofluorescence assay

The cells were inoculated in the small dish at the appropriate density and treated when the cell density reached 60%–70%. Subsequently, cells were successively fixed (4% paraformaldehyde), permeabilized (0.1% Triton X-100), blocked (0.5% bovine serum albumin), and then incubated with primary antibodies overnight at 4 °C. Finally, cells were incubated with primary antibodies followed by fluorescence-conjugated secondary antibodies (Alexa Fluor 488 [mouse] and 594 [rabbit]) of the same species in the dark at 37 °C for 1 h. After 4′,6-diamidino-2-phenylindole staining, it was washed twice with PBS, and the images were obtained under confocal microscopy (LSM780; Zeiss Microsystems; Microscope: Axio Observer.Z1/7; Objective: Plan-Apochromat 20x/0.8 M27; Acquisition software: ZEN 2.6) and quantified with ImageJ. The source of the antibodies involved in the experiment was as follows. hERG (1:50 dilution, Santa Cruz Biotechnology, sc-377388, RRID: AB_3107179; 1:100 dilution, affinity, #AF7540, RRID: AB_2843904), HDAC6 (1:50 dilution, Santa Cruz Biotechnology, sc-28386, RRID: AB_627708; 1:100 dilution, affinity, #AF6485, RRID: AB_2835165), Ub (1:50 dilution, Santa Cruz Biotechnology, sc-8017, RRID: AB_628423), LAMP1 (1:200 dilution, Santa Cruz Biotechnology, sc-20011, RRID: AB_626853), LAMP2 (1:200 dilution, Santa Cruz Biotechnology, sc-18822, RRID: AB_626858).

### Action potential detection in guinea pigs

The guinea pigs were anesthetized with sodium pentobarbital and followed by injecting heparin (1000 IU·kg^−1^), their heart was quickly taken out, and placed in Tyrode's solution containing heparin. Tyrode's solution was injected into the heart through the aorta to drain the heart congestion. The heart was immediately connected to the Langendorff perfusion system, and the uncoupling agent (−)-Blebbistatin (Sigma–Aldrich) was added to the perfusion system. After cardiac arrest, RH237 dye was added to the perfusion system. After 10 min, the dye was excited at 710 nm using a monochromatic light-emitting device. The APD signal of guinea pig heart was acquired by MiCAM05 CMOS (Sci-Media) imaging system.

### Statistical analysis

The quantitative data are expressed as “mean ± SD.” Statistical comparisons were evaluated using the Student’s *t* test or in experiments with multiple variables by ANOVA with the Tukey test to obtain indicated *p* values. Statistical significance was defined as follows: ∗*p* < 0.05, ∗∗*p* < 0.01, and ∗∗∗*p* < 0.001 was considered statistically significant and ns, not significant (*p* > 0.05).

### Ethics statement

All animal experiments adhered to the ARRIVE guidelines and were conducted in accordance with the National Research Council’s Guide for the Care and Use of Laboratory Animals. All experimental procedures involving animals were carried out according to protocols approved by the Institutional Animal Ethics Committee of Harbin Medical University (approval number: -IRB3020622).

## Data availability

All data are contained in the article and the supporting information.

## Supporting information

This article contains supporting information.

## Conflict of interest

The authors declare that they have no conflicts of interest with the contents of this article.
