# Supplementary material for: Chelerythrine triggers the prolongation of QT interval and induces cardiotoxicity by promoting the degradation of hERG channels
Source: J Biol Chem. 2024 Nov 27;301(1):108023. doi: 10.1016/j.jbc.2024.108023 (PMC11721429; doi:10.1016/j.jbc.2024.108023)
Supplement: Supporting information [file mmc1.docx]

**Supporting Information**

**Chelerythrine triggers the prolongation of QT interval and induces cardiotoxicity by promoting the degradation of hERG channels**

**Fang Wang, Baoqiang Wang, Xiwei Gu, Xiaoxu Li, Xinyu Liu, Baoxin Li***

*For corresponding: Baoxin Li, libaoxin@ems.hrbmu.edu.cn.

**Supplemental figure legend**

**Figure S1.** Survival proportions of animals.

**Figure S2.** The effects of different concentrations of CHE on QT interval and action potential duration in guinea pigs.

**Figure S3.** Representative and statistical results of hERG channel under siHDAC6 condition.

**Figure S4.** The effects of CHE on the interaction between hERG channel and HDAC6 and ubiquitination level were detected in neonatal rat cardiomyocytes (NRCMs).

**Figure S5.** PKC inhibitor bisindolylmaleimide I (BIM-1) was incubated for 24h to determine the expression of related proteins.

**
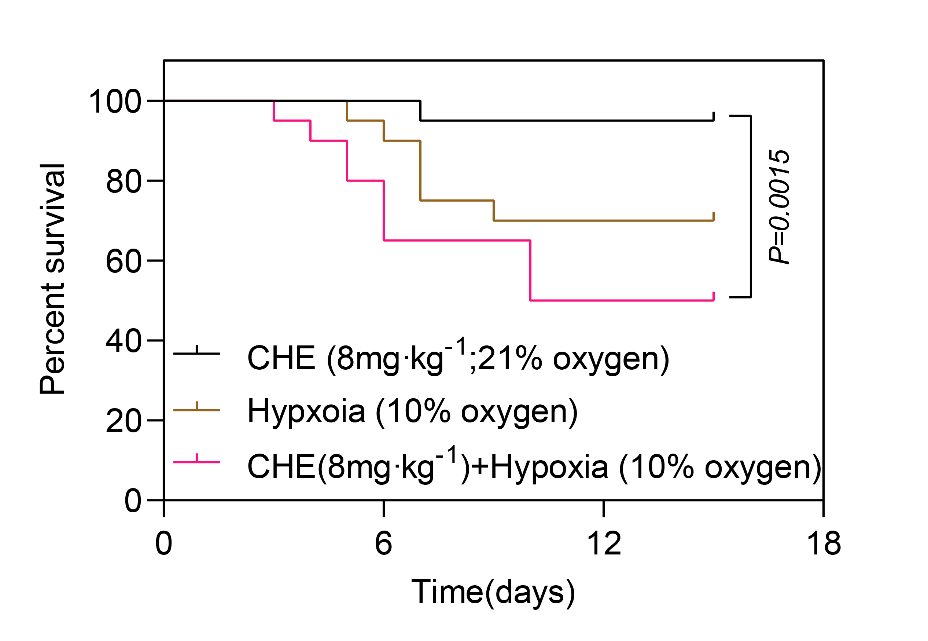
Figure S1**

**Figure S1.** **Survival proportions of animals.**

**
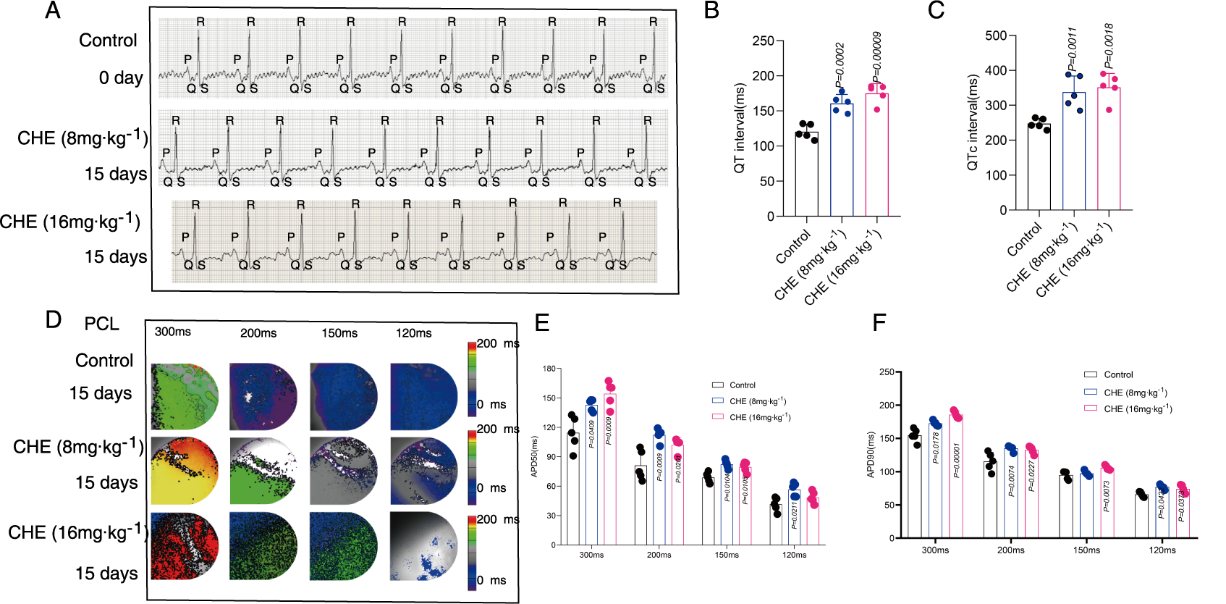
Figure S2**

**Figure S2.** **The effects of different concentrations of CHE on QT interval and action potential duration in guinea pigs.** *A,* Electrocardiogram of guinea pigs. *B-C,* Effects of CHE on QT/QTc interval in guinea pigs (n=5). *D,* Representative map of optical mapping of guinea pigs. *E-F,* Effects of CHE on APD50 and APD90 in guinea pigs (n=5). The data presented here were representative of five independent experiments.

**Figure S3**

**
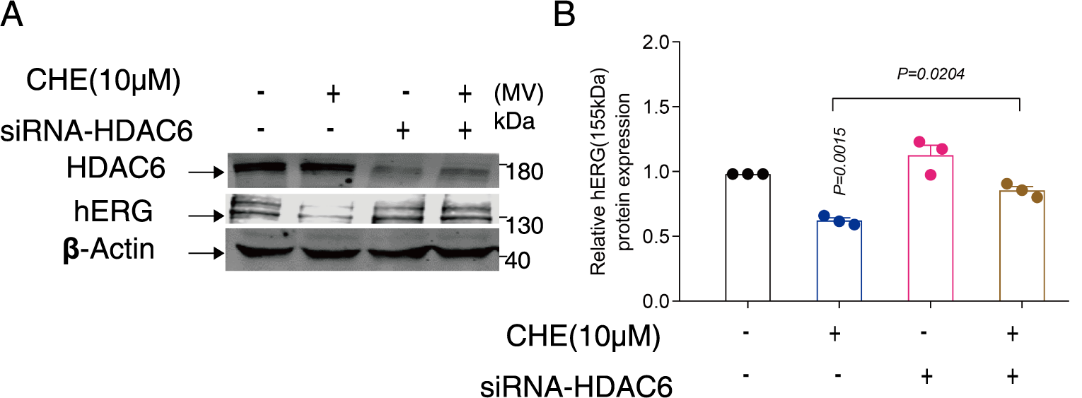
**

**Figure S3.** (*A*)Representative and (*B*)statistical results of hERG channel under siHDAC6 condition(n=3). The data presented here were representative of three independent experiments.


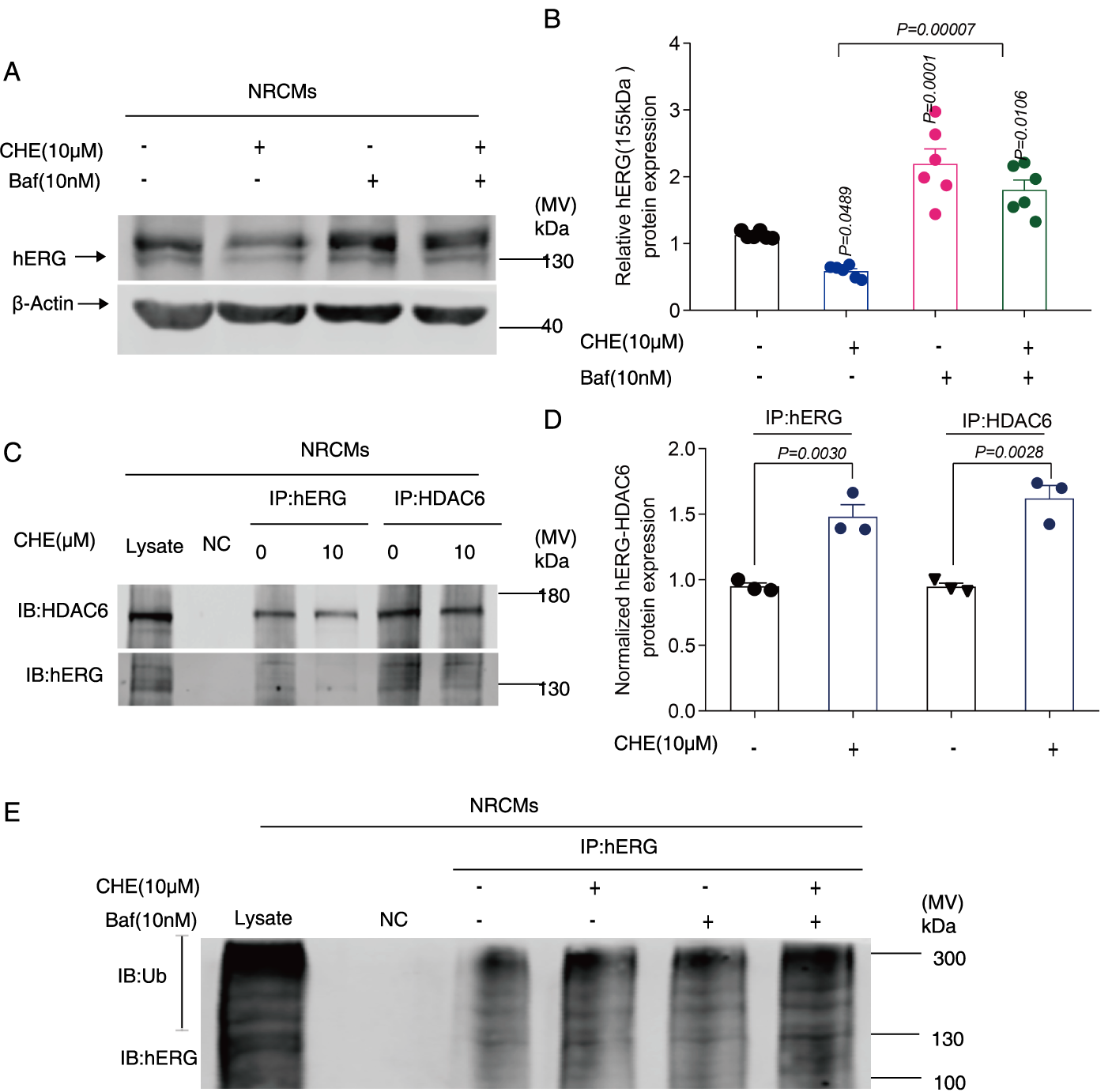
**Figure S4**

**Figure S4. The effects of CHE on the interaction between hERG channel and HDAC6 and ubiquitination level were detected in neonatal rat cardiomyocytes (NRCMs).** *A-B*, Lysosomal inhibitor Baf on CHE induced hERG protein were measured by Western blot followed(n=6). *C-D*, Interaction between HDAC6 and hERG channel by Immunoprecipitation assay(n=3). *E*, Immunoprecipitation assay for the binding of ubiquitin molecules to hERG channels (n=4). The data presented here were representative of a minimum of three independent experiments.

**Figure S5**


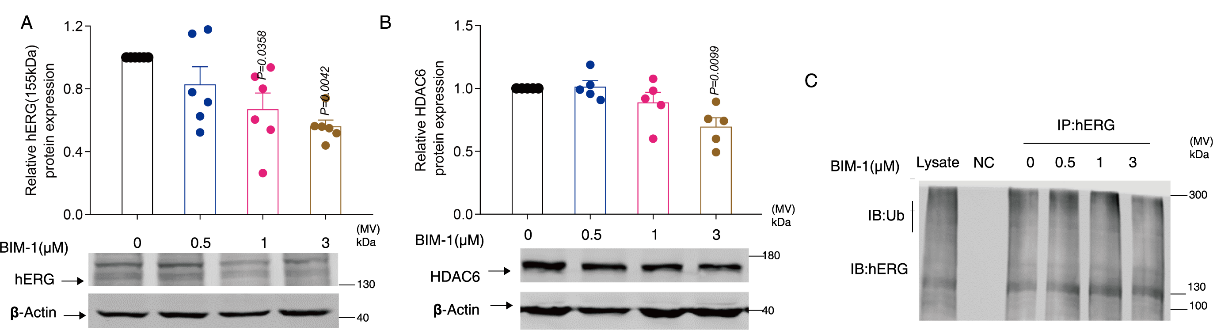


**Figure S5.** **PKC inhibitor bisindolylmaleimide I (BIM-1) was incubated for 24h to determine the expression of related proteins.** *A*, hERG protein and (*B*) HDAC6 protein were measured by Western blot followed(n=5-6). *C*, Immunoprecipitation assay for the binding of ubiquitin molecules to hERG channels (n=4). The data presented here were representative of a minimum of four independent experiments.
